# Structural Equation Modeling of Genetic and Residual Covariance Matrices for Multiple-Trait Evaluation in Beef Cattle

**DOI:** 10.3390/ani16050817

**Published:** 2026-03-05

**Authors:** Marcos Jun-Iti Yokoo, Gustavo de los Campos, Vinícius Silva Junqueira, Fernando Flores Cardoso, Guilherme Jordão Magalhães Rosa, Lucia Galvão Albuquerque

**Affiliations:** 1 Embrapa Southeastern Livestock (CPPSE), Brazilian Agricultural Research Corporation (Embrapa), Rodovia Washington Luis, km 234, São Carlos 13560-970, SP, Brazil; 2 Department of Epidemiology & Biostatistics, Statistics & Probability, Institute for Quantitative Health Sciences and Engineering, Michigan State University, East Lansing, MI 48824, USA; gustavoc@msu.edu; 3 Bayer Crop Science, Bayer, Uberlândia 38407-049, MG, Brazil; viniciussilva.junqueira@bayer.com; 4 Embrapa South Livestock (CPPSUL), Brazilian Agricultural Research Corporation (Embrapa), Rodovia BR-153, km 632,9, Bagé 16401-970, RS, Brazil; fernando.cardoso@embrapa.br; 5 Department of Animal and Dairy Sciences, University of Wisconsin, Madison, WI 53706, USA; grosa@wisc.edu; 6 Department of Animal Science, São Paulo State University (UNESP), Jaboticabal 14884-900, SP, Brazil; lgalb@fcav.unesp.br

**Keywords:** Bayesian method, factor analysis, genetic evaluation, mixed model, recursive relationships

## Abstract

The continuous growth in both the number of phenotypic records and the range of traits included in beef cattle genetic evaluations poses substantial statistical and computational challenges for the estimation of genetic and residual (co)variance matrices required for breeding value estimation. Structural equation models (SEM), implemented using either factor analysis (FA) or recursive model (REC) structures, provide a flexible framework to model genetic and residual (co)variance matrices while yielding more parsimonious and computationally efficient parameterizations. Here, SEM was applied to estimate parameters for growth and ultrasound-measured carcass traits in beef cattle. The dataset comprised 2942 animals, and six traits were evaluated using standard multiple-trait mixed models (SMTM) and SEM. We considered FA and REC models implemented with six alternative parameterizations, in which random effects were represented as linear combinations of fewer unobservable random variables. Relative to the SMTM, both the model with two factors in the genetic covariance matrix (FA2G) and the model in which six recursive effects were constrained to zero in the residual covariance matrix (REC1) demonstrated a strong ability to capture genetic variability, as reflected by comparable heritability estimates. Correlations between estimated breeding values (EBV) for the same traits across models were consistently high, ranging from 0.94 to 1.00, indicating strong agreement among model estimates. The FA2G model was the most parsimonious in terms of the effective number of parameters ($p_{D}$), with 431.2 $p_{D}$, corresponding to a reduction of 25.3 parameters relative to the SMTM. The REC1 model also emerged as a competitive alternative for this dataset, exhibiting a lower $p_{D}$ (443.6) than the SMTM (456.5) and the most favorable deviance information criterion among all models evaluated (e.g., 37,868.6 for REC1 versus 37,874.7 for SMTM). Overall, these results demonstrate that mixed-effects multi-trait models for beef cattle genetic evaluation can be effectively implemented using FA or REC structures, which provide parsimonious representations of the underlying covariance patterns while maintaining high agreement in EBV.

## Simple Summary

In beef cattle, farmers collect vast amounts of data on many different traits that serve as selection criteria. Therefore, genetic breeding programs must estimate the breeding values by correcting for the relationships that affect the traits throughout the course of causality. For example, a measurement of a particular trait collected at an earlier age may influence another trait that will be collected at a later age. Estimating how these traits are transmitted genetically becomes increasingly complex as the number of traits and records increases. Farmers select animals for multiple traits. Therefore, breeding programs must consider the relationships among these traits. This study aimed to evaluate simpler mathematical methods, known as structural equation models, to assess their accuracy compared to traditional evaluation methods. Data from beef cattle were analyzed, looking at growth and carcass traits. The results showed that some new models are just as accurate as traditional ones in identifying the best animals for breeding. Furthermore, the causal relationships between the various traits could be identified, aiding in the selection and decision-making processes, helping farmers be more efficient and productive for society.

## 1. Introduction

Standard multiple-trait mixed models (SMTM) are widely used in genetic evaluations to estimate unstructured (co)variance matrices across multiple traits [1]. Under an unstructured (co)variance specification, all variances and covariances are freely estimated without imposing mathematical or biological constraints, and no assumptions are made regarding relationships among traits beyond symmetry and positive definiteness. However, the number of (co)variance parameters increases quadratically as the number of traits increases, and high phenotypic correlations may result in near-singular genetic and residual covariance matrices, thereby hindering algorithm convergence and compromising the reliability of statistical inference [1,2,3,4].

Structural equation models (SEM) are often used to obtain more parsimonious (and sometimes more interpretable) parametrization of multivariate models. This approach has been used in quantitative genetics to structure the genetic and environmental covariance matrices of multivariate linear mixed models [1,2,3,4,5,6,7,8], commonly used for genetic evaluations. This is primarily due to its ability to model causal relationships among latent variables, allowing for the modeling of complex phenomena while simultaneously reducing the dimensionality of the data [2,3,6,9]. SEM methodology comprises many different techniques and procedures used together to model covariance structures. These models represent a natural extension of the multivariate linear mixed modeling framework described by Henderson and Quaas [10] through the imposition of structured covariance parameterizations that can express functional networks among traits [11]. Gianola and Sorensen [5] investigated the application of recursive (REC) and simultaneous equation models, both of which are special cases of SEM, applied to phenotypes. De los Campos et al. [2] proposed a methodology to search for recursive and simultaneous effects among phenotypes within a multivariate linear mixed model, which has been applied to various species and traits [2,4]. Some authors discussed the use of REC models operating on genetic covariance matrices [6,12,13]. In certain cases, explicit modeling of covariance matrices facilitates the inference and analysis of causal relationships among breeding values of multiple traits or the evaluation of functions (e.g., ratios) of correlated traits, thereby enabling a reduction in model dimensionality through more parsimonious and efficient parameterizations [4,6], without compromising the accuracy of breeding value estimates [14].

Alternatively, factor analysis (FA), another specific case of SEM, can be used to reduce the dimension of the estimated genetic covariance matrix [3,7] and achieve a more parsimonious model of genetic effects within a multivariate linear mixed model, without decreasing the original number of traits and records. In the FA framework, the observed traits are modeled as linear combinations of fewer unobservable latent factors and trait-specific residual components, effectively capturing the common sources of variation underlying correlated traits while preserving individual trait identities. This approach has been successfully applied in quantitative genetics to structure genetic and environmental covariance matrices [3,8], proving particularly useful when analyzing highly correlated traits where traditional unstructured covariance matrices may suffer from convergence issues or poor estimability. The reduction in the number of parameters not only improves computational efficiency but can also lead to more stable parameter estimates and better model convergence, especially in datasets with limited sample sizes relative to the number of traits being analyzed [3]. Moreover, FA models can provide biological insights by identifying latent factors that may represent underlying biological processes or developmental pathways common to multiple phenotypes.

While previous studies have demonstrated the utility of SEM in various contexts, their application to structuring both genetic and residual covariance matrices independently in beef cattle breeding remains underexplored. In the current omics era, breeding programs are increasingly collecting larger numbers of correlated phenotypes, making computationally efficient, statistically robust methods for multi-trait genetic evaluation even more critical. These more parsimonious models offer practical advantages for routine genetic evaluations in breeding programs, enabling the simultaneous analysis of multiple traits across large populations with improved computational efficiency and numerical stability compared to fully parameterized models. We hypothesized that two different SEMs could be applied simultaneously and independently to the genetic and residual covariance matrices with a comprehensive accuracy using real beef cattle breeding data. Thereby, this study aimed to evaluate the performance of factor analysis and recursive models, applied independently to genetic and residual covariance matrices for growth and ultrasound-measured carcass traits in Nellore cattle, and to compare them with standard multiple-trait mixed models in terms of parameter estimation, breeding value estimation, and model fit.

## 2. Materials and Methods

### 2.1. Dataset Description

**Figure 1:**
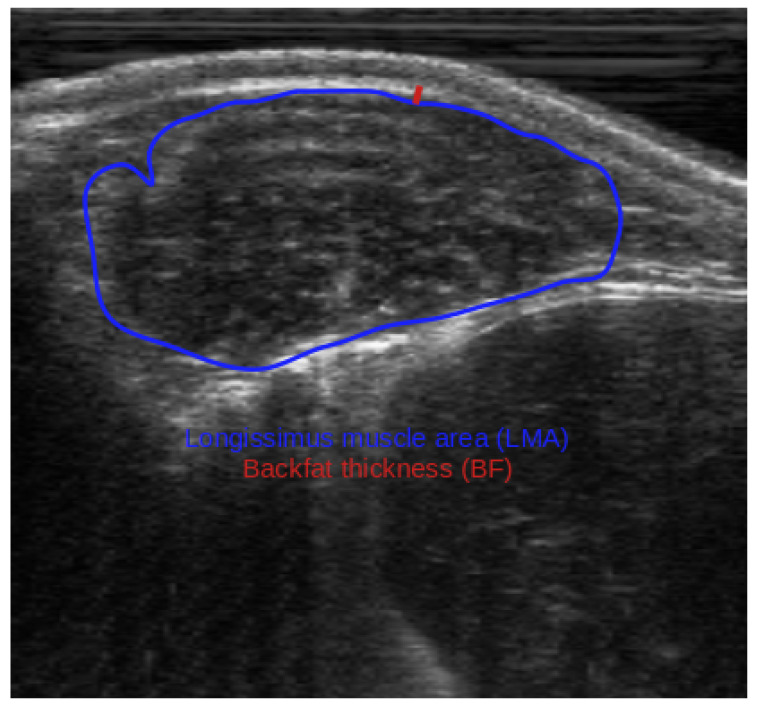
Example of a traced ultrasound image illustrating LMA (blue line) and BF (red line) in Nellore cattle.

**Figure 2:**
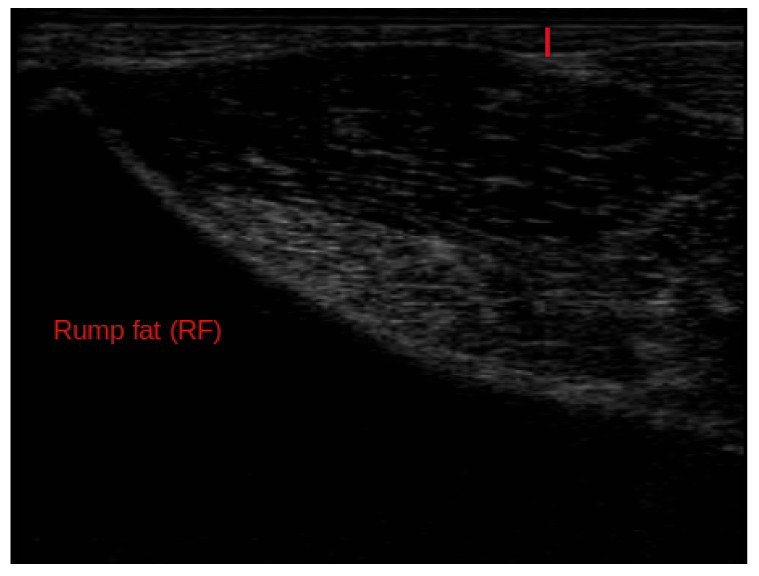
Example of a traced ultrasound image illustrating RF (red line) in Nellore cattle.

This research used records from 2942 Nellore cattle collected between 2004 and 2006. The data came from an established database comprising 10 herds enrolled in the Brazilian Nellore Breeding Program across six states. On-farm data collection was performed under standardized management protocols and in accordance with the guidelines of the National Research Council (NRC, [15]). To obtain carcass-related phenotypes without slaughtering the animals, real-time ultrasound technology was used, as described by Duff et al. [16] and Meškinytė et al. [17]. Real-time ultrasound carcass data included longissimus muscle area (LMA, cm^2^, Figure 1), backfat thickness (BF, mm, Figure 1), and rump fat thickness (RF, mm, Figure 2). Additional traits recorded at the same time included body weight (BW, kg) and hip height (HH, cm), as well as scrotal circumference adjusted to 450 days of age (SC, mm). Scrotal circumference adjusted to 450 days of age was obtained as described in the Guidelines for Uniform Beef Improvement Programs [18]. The traits were assessed in animals aged between 480 and 629 days of age.

Contemporary groups (CGs) were defined as animals of the same sex that were born in the same herd, year, and season (dry or rainy) and managed under identical rearing conditions until data recording. A total of 302 contemporary groups were formed, with an average of 9.7 animals per group (range: 3 to 45 animals). Additional effects considered in the analyses included age of animal at scanning (linear covariate for all traits except SC) and age of dam at calving (linear and quadratic covariates for BF, RF, HH, and BW; classes for LMA and SC). A description of the traits and data structure for Brazilian Nellore cattle is presented in Table 1.

### 2.2. Standard Multi-Trait Mixed Model

All models were evaluated using Bayesian inference implemented via Gibbs sampling. In the sections that follow, we first describe the SMTM commonly used in genetic evaluations, and subsequently present model extensions incorporating FA and REC structures.

In the SMTM, a sire model was fitted for *p* traits measured on *n* individuals. The mixed-model equation can be described as:

(1) y=Xβ+Zu+ϵ,

where y is the vector of phenotypic observations, β is the vector of fixed effects, u is the vector of random additive sire effects, ϵ is the vector of residual effects, and X and Z are the corresponding incidence matrices. Under the Bayesian framework, the likelihood function was specified as:

$$y|\beta ,u,G_{0},R_{0}∼N\left(X\beta +Zu,I_{n}⊗R_{0}\right),$$

with prior distributions $u|A,G_{0}∼N\left(0,A⊗G_{0}\right)$ and $\epsilon |R_{0}∼N\left(0,I_{n}⊗R_{0}\right)$, where $G_{0}$ is the p×p additive genetic (co)variance matrix among sire effects, $R_{0}$ is the p×p residual (co)variance matrix, A is Wright’s numerator relationship matrix among sires, $I_{n}$ is an identity matrix of order *n*, and ⊗ denotes the Kronecker product operator.

Prior distributions for model parameters were specified as follows. Systematic effects included contemporary groups, animal age at scanning (linear effect, except for SC), and dam age (linear and quadratic effects for BF, RF, HH, and BW). For the vector of systematic effects β with vague information, prior distributions were assigned as:

$$\beta ∼N(0,I⊗100^{2}),$$

For the (co)variance matrices, scaled inverse Wishart distributions were assigned as prior distributions:

$$G_{0}∼\mathrm{IW}\left(S_{g},\nu_{g}\right)\;\mathrm{and}\;R_{0}∼\mathrm{IW}\left(S_{e},\nu_{e}\right),$$

where $S_{g}$ and $S_{e}$ are p×p scale matrices and $\nu_{g}$ and $\nu_{e}$ are degrees of freedom parameters. The joint posterior distribution of all model parameters is proportional to the product of the likelihood and prior distributions:

$$p\left(\beta ,u,G_{0},R_{0}|y\right)\propto p\left(y|\beta ,u,G_{0},R_{0}\right)\times p\left(u|A,G_{0}\right)\times p\left(\beta \right)\times p\left(G_{0}\right)\times p\left(R_{0}\right).$$

### 2.3. Factor Analytic Model

Factor analysis is a multivariate statistical technique that simplifies complex and correlated data structures [19,20]. It can be viewed as an extension of principal component analysis, where the factor-analytic variance-covariance structure serves as a parsimonious approximation to a fully unstructured variance-covariance matrix, enabling more efficient parameterization of complex correlation patterns. In a standard FA, a vector of random variables (e.g., u, representing breeding values) is decomposed into a linear combination of a reduced number of unobservable random variables called common factors (f), with an unobservable incidence matrix (Λ) of factor loadings, plus a vector of trait-specific errors (δ) to each individual *i* that represent the lack of fit of the model. In compact notation:

(2) $$u_{i}=\Lambda_{u}f_{i}+\delta_{u,i},$$

In vector notation, the factor analytic model for these effects can be written as:

(3) $$u=(I_{n}⊗\Lambda )f+\delta ,$$

where $u={\left(u_{1}^{\prime },\ldots ,u_{n}^{\prime }\right)}^{\prime }$, $f={\left(f_{1}^{\prime },\ldots ,f_{n}^{\prime }\right)}^{\prime }$, and $\delta ={\left(\delta_{1}^{\prime },\ldots ,\delta_{n}^{\prime }\right)}^{\prime }$. This results in the following covariance matrix of u:

$$\mathrm{Cov}\left(u_{i}\right)=G_{0}=\Lambda_{u}\Lambda_{u}^{\prime }+\Psi_{u},$$

where $\Psi_{u}=\mathrm{Diag}\left\{\psi_{u,1},\ldots ,\psi_{u,p}\right\}$ is a diagonal matrix of specific variances. Likewise, the residual vector (ϵ) can be decomposed into common and trait-specific factors. Consequently, the marginal distributions of $u_{i}$ and $\epsilon_{i}$ are:

$$u_{i}\overset{\mathrm{iid}}{∼}N\left(0,\Lambda_{u}\Lambda_{u}^{\prime }+\Psi_{u}\right)\;\mathrm{and}\;\epsilon_{i}\overset{\mathrm{iid}}{∼}N\left(0,\Lambda_{\epsilon }\Lambda_{\epsilon }^{\prime }+\Psi_{\epsilon }\right),$$

where “iid” stands for “independent and identically distributed”.

To model the additive genetic and residual (co)variance matrices using FA, it was assumed that Equation (2) holds for the vector of random additive genetic effects (u) in Equation (1) and, likewise, for the vector of random residual effects (ϵ) such that:

(4) $$y=X\beta +Z\left[\left(I_{u}⊗\Lambda_{u}\right)f_{u}\right]+Z\delta_{u}+\left[\left(I_{\epsilon }⊗\Lambda_{\epsilon }\right)f_{\epsilon }\right]+\delta_{\epsilon },$$

where Λ, X, Z and β are as before and f and δ are interpreted as vectors of common and specific additive genetic effects, respectively, similarly for the random residual effects (ϵ). Combining the assumptions of the FA model described above with those of SMTM leads to the following random effects joint distribution,

$$p\left(\epsilon ,u\right)=N\left[\epsilon |0,I_{\epsilon }⊗\left(\Lambda_{\epsilon }\Lambda_{\epsilon }^{\prime }+\Psi_{\epsilon }\right)\right]\;N\left[u|0,A⊗\left(\Lambda_{u}\Lambda_{u}^{\prime }+\Psi_{u}\right)\right],$$

where $\Lambda_{u}$ and $\Lambda_{\epsilon }$ are the matrices of additive genetic and residual factor loads, respectively, $\Psi_{u}$ and $\Psi_{\epsilon }$ are the diagonal matrices of specific additive genetic and residual variances, respectively. More details on the implementation of this model can be found in [8].

### 2.4. Recursive Models

Recursive models are a category of SEMs that postulate causal and unidirectional relationships between latent variables, unlike simultaneous models, which admit mutual feedback effects between traits. For more information on the procedure for REC effects decomposition, see [2]. Using REC, the vector u can be decomposed as:

(5) $$u_{i}=\Pi_{u}u_{i}+{£}_{u,i},$$

where $u_{i}$ is the random effect of the *i*th sire, $\Pi_{u}=\left\{\pi_{u,ij}\right\}$ is a strictly lower-triangular matrix; i.e., $\pi_{u,ij}=0$ for all i≤j, whose non-zero entries define recursive effects, and ${£}_{u,i}$ is a vector of random effects whose (co)variance matrix is: $\mathrm{Cov}\left({£}_{u}\right)=A⊗\Gamma_{u}$ where $\Gamma_{u}=\mathrm{Cov}\left({£}_{u,i}\right)$ is a diagonal matrix. The reduced form model of Equation (5) is:

(6) $$u_{i}={\left(I-\Pi_{u}\right)}^{-1}{£}_{u,i},$$

Similarly to the FA model, to implement the REC to model the additive genetic and the residual (co)variance matrices, it was assumed that Equation (6) holds for the vector of random additive genetic effects (u) in Equation (1), likewise for the vector of random residual effects (ϵ). Therefore, the additive genetic covariance matrix is equal to $G_{0}$, as $\mathrm{Cov}\left(u_{i}\right)=G_{0}={\left(I-\Pi_{u}\right)}^{-1}\Gamma_{u}{\left(I-\Pi_{u}\right)}^{-1\prime }$. Similarly, using REC for residuals, $R_{0}$ can be represented as $\mathrm{Cov}\left(\epsilon_{i}\right)=R_{0}={\left(I-\Pi_{\epsilon }\right)}^{-1}\Gamma_{\epsilon }{\left(I-\Pi_{\epsilon }\right)}^{-1\prime }$, where $\Pi_{\epsilon }$ is a strictly lower-triangular matrix defining recursive effects between model residuals, and $\Gamma_{\epsilon }$ is diagonal. A fully recursive model (FRM) occurs when all lower-triangular entries of $\Pi_{u}$ and of $\Pi_{\epsilon }$ are non-zero, i.e., all $\pi_{u,ij}$ and $\pi_{\epsilon ,ij}$ with i>j are parameters to be estimated. This model has as many (co)dispersion parameters as the SMTM, and there is a one to one map from the unknowns in $\left\{\Pi_{u},\Gamma_{u},\Pi_{\epsilon },\Gamma_{\epsilon }\right\}$ to those in $\left\{G_{0},R_{0}\right\}$. Therefore, the FRM is just a re-parameterization of SMTM; however, various degrees of parsimony can be obtained by setting some of the lower-triangular entries of $\Pi_{u}$ and $\Pi_{\epsilon }$ to zero. Consequently, the models evaluated in this study differed in the structural specification of the genetic and residual (co)variance matrices, with distinct sets of constraints imposed on each, as detailed below.

### 2.5. Statistical Analyses

Bayesian inference and MCMC implementation: All genetic parameters were estimated using a Bayesian inference framework implemented via Gibbs sampling, as performed using the MTM package [21] in the R statistical computing environment [22]. An initial chain of 1,500,000 iterations was run to assess convergence. The final chain length, burn-in period, and thinning interval were determined using the criteria of [23], implemented in the Bayesian Output Analysis (BOA) package [24]. Convergence was further evaluated through visual inspection of trace plots for variance components. Based on these diagnostics, posterior inference was based on 146,000 samples obtained after discarding the first 40,000 iterations as burn-in and retaining every 10th iteration thereafter.

Model comparison: Multiple alternative models were fitted to assess the effectiveness of parsimonious covariance structures. The SMTM with unstructured (co)variance matrices served as the baseline, requiring estimation of 42 (co)variance parameters (21 for $G_{0}$ and 21 for $R_{0}$).

Factor analytic models: Given the limited number of traits (six), only models with two or three latent factors were evaluated for each (co)variance matrix; consequently, four factor-analytic (FA) models were assessed. The FA2F model specified two common factors for both additive genetic and residual (co)variance matrices, reducing the total number of parameters to 24 (12 genetic + 12 residual). The FA3F model extended this to three factors, increasing the parameter count to 36 (18 genetic + 18 residual). To evaluate the benefit of parsimony in only one (co)variance component, two additional models were fitted: FA2G applied two factors to $G_{0}$ while maintaining the unstructured $R_{0}$ (33 parameters total), whereas FA2R applied two factors to $R_{0}$ while maintaining the unstructured $G_{0}$ (33 parameters total).

Recursive models: In total, three REC were evaluated. First, a fully recursive model (FRM) was fitted, which is equivalent to the SMTM in terms of the number of parameters but reparameterizes the covariance structures via recursive pathways. Based on the results obtained from the FRM, and with the objective of reducing the number of estimated parameters, two additional reduced models were subsequently constructed by imposing zero constraints on selected recursive effects. The REC1 model set the ijth entry of $\Pi_{\epsilon }$ to zero if $|{\hat{\pi }}_{ij}|/{\mathrm{sd}}_{ij}<1.96$ or i≤j, where ${\hat{\pi }}_{ij}$ and ${\mathrm{sd}}_{ij}$ denote the posterior mean and standard deviation of the ijth entry of $\Pi_{\epsilon }$ from FRM, respectively. This criterion resulted in six recursive effects being removed from $\Pi_{\epsilon }$. The REC2 model removed any effect in $\Pi_{u}$ or $\Pi_{\epsilon }$ whose posterior mean from FRM had absolute value less than 0.15 (an arbitrary threshold chosen to evaluate a more parsimonious structure), resulting in removal of 15 recursive effects (10 from $\Pi_{\epsilon }$ and 5 from $\Pi_{u}$).

Model selection criteria: Considering that the SMTM represents the standard benchmark for estimating genetic values, models were compared using the deviance information criterion (DIC; [25,26]), the posterior mean of the log-likelihood [Mean(L)], and the effective number of parameters ($p_{D}$; [25,26]). Lower DIC values indicate better model fit penalized for complexity, while $p_{D}$ quantifies model complexity. In addition, Spearman’s rank and Pearson’s product–moment correlations between estimated breeding values (EBV) for the same traits obtained from the SMTM and alternative models (REC and FA) were computed to evaluate the consistency of animal ranking across modeling approaches.

## 3. Results

### 3.1. Genetic Parameters

Table 2, Table 3 and Table 4 report estimates of heritabilities (diagonal elements), genetic correlations (above the diagonal), and residual correlations (below the diagonal) assessed from the SMTM, FA2G, and REC1 models, respectively. Across these models, standard errors of heritability estimates ranged between 0.05 and 0.12. Correlations of Spearman and Pearson between EBV for the same traits across models were consistently high, ranging from 0.94 to 1.00.

For the genetic correlations, the standard errors obtained by these three models presented in Table 2, Table 3 and Table 4 ranged from 0.07 to 0.23. Considering the overlap within ±1.0 standard errors, differences between genetic correlation estimates obtained by SMTM (Table 2) and the REC1 (Table 4) model were negligible. In contrast, discrepancies between the SMTM (Table 2) and FA2G (Table 3) models were observed for LMA and BW, BF and RF, BF and HH, RF and HH, and BW and HH.

For the residual correlations, the standard errors obtained by these three models presented in Table 2, Table 3 and Table 4 ranged from 0.01 to 0.07. Considering these standard error ranges, differences in residual correlation estimates obtained with the SMTM (Table 2) and REC1 (Table 4) models were observed only for SC and BF. The differences between the residual correlation estimates obtained by the SMTM (Table 2) and FA2G (Table 3) models were not significant.

### 3.2. Recursive Effects

The ordering of traits within the model directly determines the identifiability of causal effects. For example, a recursive effect of adult weight acting on weaning weight for the same individual would likely not be identifiable. Because all traits analyzed in this study were recorded on the same day, their expression was observed simultaneously, which facilitates the specification of admissible causal relationships. Accordingly, estimates of genetic and residual recursive effects obtained from the FRM in Nellore cattle are presented in Table 5, under the assumption that causal effects flow from left to right (i.e., from the trait in the first column to subsequent traits). A variable may exert or receive causal influence (directly or indirectly) from another variable that precedes it in the ordering; however, such relationships represent simultaneous effects rather than recursive effects and are therefore not considered in the present study.

### 3.3. Model Comparison

The DIC, the Mean(L), and the $p_{D}$ used to compare the proposed models are shown in Table 6. Although the SMTM model is considered fully parameterized for the additive genetic and residual matrices, the $p_{D}$ were largest in models that have many (co)dispersion parameters, as in the FA3F and FA2R, compared to the SMTM model; thus, the model with the smallest $p_{D}$ was FA2G.

The models with the best (smallest) DIC and Mean(L) were REC1 and SMTM, respectively. Conversely, FA3F was the worst model for these three comparison criteria (DIC, Mean(L), and $p_{D}$).

## 4. Discussion

This study aimed to evaluate the potential advantages of using structured equation models, such as recursive and factor-analytic models, for sire modeling in beef cattle genetic evaluations of ultrasound carcass and growth traits. Given the magnitude of standard errors, differences between heritability estimates obtained by SMTM and the two best models (REC1 and FA2G) were only observed for BF and RF traits in the FA2G model, which yielded slightly lower estimates, except for SC in the REC1 model, while exhibiting the same pattern and remaining within one standard deviation. The EBVs estimated under the different models were highly consistent, with Pearson and Spearman correlation coefficients approaching 1.0 for each trait, indicating that selection decisions would be essentially unchanged using any of the methodologies considered here, despite the lower genetic variability (heritability estimates) of some traits in some models. Under a genetic evaluation framework adopting these SEM approaches to estimate breeding values, the corresponding variance components would be fixed using the Best Linear Unbiased Prediction (BLUP). Overall, the SMTM more effectively captured the heritability estimates for carcass traits measured by ultrasound (LMA, BF, and RF), as reported in Table 2, compared with the corresponding estimates presented in Table 3 and Table 4. In contrast, for the remaining growth-related traits (BW, HH, and SC), the REC1 model yielded superior performance in estimating heritabilities. This information is important for guiding the choice of the genetic evaluation model, with the objective of maximizing selection accuracy. Moreover, these estimates are consistent with those reported in previous studies [16,27].

The FA2G model estimated genetic correlations with the same pattern as the SMTM model, but with a lower magnitude. High genetic correlations among traits imply a genetic covariance matrix that is close to rank deficient, which often results in convergence difficulties, imprecise estimation of factor loadings, and redundancy among latent factors [3,19]. Consequently, FA models are expected to provide reliable approximations of the underlying covariance structure primarily when genetic correlations are of moderate or low magnitude, as discussed by [28]. Conversely, most genetic correlations estimated using the REC1 model were of slightly greater magnitude than those obtained with the SMTM, but yet very similar, indicating a good alternative for modeling additive genetic covariance matrices in beef cattle.

As expected, the residual covariance matrices estimated under the REC1 and FA2G models closely resembled those obtained under the SMTM, except for the association between SC and BF in the REC1 model. Nevertheless, the residual correlation in the REC1 model was basically null (0.09±0.01). De los Campos et al. [2] likewise did not observe significant changes in covariance estimates when applying REC or simultaneous models. Considering the reasonable agreement between covariance parameters and breeding values estimated by the REC1 and FA2G models with the SMTM, it could be suggested that these structural equations can be successfully used to reduce dimensionality of genetic evaluations, optimizing processing by a single analysis including all traits of interest and, consequently, providing faster and reliable estimation of breeding values compared to the traditional univariate analyses for each selection criteria.

Recursive models act separately on u and ϵ, allowing specific patterns to be captured in each component (Table 3). Differences in the patterns of genetic and residual correlations can be interpreted in light of the estimated recursive effects, and based on this information, irrelevant effects can be suppressed from the covariance matrices [2,5,6,12]. The six recursive effects zeroed out in $\Pi_{\epsilon }$ for the REC1 model were between RF and LMA, SC and LMA, BW and RF, HH and RF, SC and RF, and SC and HH. Consequently, the REC1 model excluded six recursive effects that were effectively negligible among the 21 parameters initially considered, indicating that these effects do not materially contribute to the estimation of breeding values for the traits involved. This likely reflects the fact that these residual causal effects do not operate at the genetic level nor directly influence trait expression. These results suggest that, when residual causal effects are close to zero, this class of SEM can facilitate the joint estimation of breeding values for multiple traits within a single analytical framework.

In terms of the $p_{D}$ criterion, all recursive models (FRM, REC1, and REC2) had smaller $p_{D}$ values than the SMTM model; even for FRM, which has the same dimension as the SMTM, some benefits were observed when using this kind of structural equation model. For the FA models, $p_{D}$ favored the FA2G (two factors) model over all other FA and REC models. However, when we tested a model with three factors (FA3F), the $p_{D}$ was the largest. This was expected due to the number of parameters (*p*) of $\mathrm{Cov}\left(u\right)=G_{0}$ in the FA, which can be calculated as p=q+mq−m(m−1)/2 parameters, where q×m are the dimensions of the Λ matrix of factor loads (Equation (2)). FA3F has 21 *p* in each matrix (additive genetic and residual), while FA2F has 17 *p*. In the SMTM, the *p* of $G_{0}$ can be calculated as p=q(q+1)/2 parameters, where q×q are the dimensions of the $G_{0}$ matrix, thus yielding 21 *p* in each matrix (additive genetic and residual) in the SMTM model. Depending on the data set, i.e., the number of records, a greater number of traits, and the most correlated traits, the FA models may be used as a special case of SEM to reduce the covariance matrices’ rank in the model and provide a more parsimonious estimation of genetic parameters compared to SMTM. In this paper, the FA3F model had a poorer fit to the data than the other models because this dataset has only six traits. Nevertheless, if the dataset had more traits, a model with more factors would probably work better at reducing the $p_{D}$. Normally, increasing the number of factors in the model improves the representation of relationships among traits; however, when the number of traits is small, only a limited number of factors can be reliably estimated. In the present study, the analysis was restricted to six traits, and the number of records for SC was limited because this trait is recorded only in males (n=1340). This could at least partially explain the worst performance in terms of DIC, $p_{D}$, and Mean(L) of the FA3F model. As discussed by [28,29], the FA model usually reduces the rank of covariance matrices. Still, the Ψ matrix has to be close to zero $\mathrm{Cov}\left(u\right)=G_{0}=\Lambda \Lambda^{\prime }+\Psi$, when specific effects are assumed absent. This leads to a mixed-model formulation characterized by covariance matrices of reduced rank.

In the same sense, the Mean(L) and DIC demonstrated considerably better fits to the data for the models SMTM and REC1 than for all the other FA and REC models. Since DIC also accounts for dimensionality through a penalty ($p_{D}$), the REC1 model may represent a viable alternative to the SMTM for beef cattle genetic evaluation procedures involving multi-trait analyses.

In general, even though FA2G was effective in terms of $p_{D}$, comparing all the criteria and parameters, the REC1 model proved to be a suitable alternative modeling approach for this type of dataset (e.g., six traits) because it had a smaller $p_{D}$ compared to the SMTM model, better DIC, and a reasonable Mean(L) compared with all other models tested. This model, when applied to the residual effect, can be used to obtain more parsimonious representations of the residual covariance matrix. This reduces the number of parameters to be estimated, and may provide useful insight into the underlying patterns of (co)variation. Using complex traits (i.e., ratio), Jamrozik et al. [4] also demonstrated the advantages of applying REC models in multiple-trait evaluation. The results of the model comparison criteria demonstrate that growth and ultrasound-measured carcass traits in beef cattle can be effectively analyzed using REC for the estimation of breeding values and the inference of putative causal relationships among latent variables, allowing the representation of complex biological processes while simultaneously reducing the dimensionality of the data.

Breeding values estimated under FRM or REC differ conceptually from those obtained under SMTM. Under FRM or REC, breeding values represent direct genetic effects on trait expression without accounting for causal genetic effects from other traits included in the model. In contrast, breeding values derived from SMTM reflect total genetic effects, incorporating potential causal relationships among traits. Consistent with this interpretation, analysis of the biological meaning and causal structure of the recursive relationships estimated in the genetic matrix (Table 5) reveals a strong genetic influence of BW on HH. This result is biologically plausible, as many genes influencing BW also affect frame size [27], for which HH serves as an indicator trait. The resulting genetic architecture poses a challenging selection scenario: because BW and HH are positively correlated, achieving simultaneous gains in BW while maintaining or reducing HH is difficult. Moderate causal effects were also detected of BF on RF, indicating heterogeneity in subcutaneous fat deposition; and of LMA on BF, BW, and HH, suggesting that unfavorable genetic potential for muscle development adversely affects fat deposition, body weight, and skeletal growth. To maximize genetic gain and improve selection accuracy across multiple traits, the use of selection indices represents an effective alternative [30].

For the recursive relationships estimated in the residual matrix from the FRM (Table 5), moderate effects are observed of RF to BF; of BW to LMA; of HH to BW, and of SC to BW. These residual-level relationships indicate that environmental or management factors affecting one trait may exert correlated effects on others.

From a quantitative genetics perspective, RECs provide a coherent framework for representing causal relationships among traits because they enable explicit modeling and interpretation of the biological mechanisms underlying trait expression. Their routine use, however, is constrained by high computational demands that increase with model dimensionality and by the need for reliable prior knowledge of the causal structure [6,12], as misspecification may lead to biased estimates and misleading genetic inferences (e.g., assuming a causal effect of adult weight on weaning weight). Robust estimation further requires high-quality phenotypic records collected on the same individuals, with a large number of measurements, even if it is a trait measured in only one sex, such as SC. Despite these limitations, the models evaluated here showed satisfactory performance. Further studies involving more traits and breeds are warranted, particularly to assess computational efficiency. A key advantage of the SEM approach in this study is its capacity to specify structural assumptions independently for the genetic and residual (co)variance matrices, using either REC or FA parameterizations. The REC structure enables the estimation of genetic causal effects independently of residual covariances and allows the exclusion of negligible causal pathways, whereas the FA structure facilitates breeding value estimation by reducing the dimensionality of the genetic covariance matrix relative to fully unstructured models, provided that model fit is improved.

## 5. Conclusions

The results of this study demonstrate that growth and ultrasound-measured carcass traits in Nellore beef cattle can be effectively analyzed using factor-analytic and/or recursive models applied independently to the genetic and residual covariance matrices, enabling the joint estimation of breeding values for multiple traits within a single analytical framework. Depending on the size of the dataset, particularly the number of records and traits, factor analytic and recursive structures, applied either independently or jointly, can substantially reduce the effective rank of the covariance matrices and yield more parsimonious and stable estimates of genetic parameters than standard multiple-trait mixed models, especially when covariances among traits are of low magnitude.

## Figures and Tables

**Table 1:** Descriptive statistics of ultrasound carcass and growth traits in Nellore cattle.

Traits	No. of Records	Mean ± SE	No. of Sires	No. of Dams	Quantity of CGs
LMA	2770	48.05 ± 8.36	231	2552	243
BF	2577	1.87 ± 1.07	226	2397	253
RF	2566	2.95 ± 1.94	226	2384	252
SC	1340	245.87 ± 30.22	106	1009	88
HH	2349	136.06 ± 5.04	226	2308	250
BW	2942	339.69 ± 65.98	236	2683	302

LMA = longissimus muscle area (cm^2^); BF = backfat thickness (mm); RF = rump fat thickness (mm); SC = standardized scrotal circumference (mm) at 450 days of age; BW and HH = body weight (kg) and hip height (cm), respectively, obtained at the time of scanning. SE = standard deviation; Quantity of CGs = amount of contemporary groups within each trait.

**Table 2:** Estimates of heritability (diagonal), genetic (above diagonal) and residual (below diagonal) correlations, with standard errors, from the SMTM.

Traits	LMA	BF	RF	BW	HH	SC
LMA	0.29±0.06	0.13±0.15	0.05±0.16	0.35±0.13	−0.14±0.15	0.19±0.17
BF	0.14±0.02	0.47±0.10	0.62±0.10	0.03±0.17	−0.52±0.12	0.09±0.22
RF	0.11±0.02	0.57±0.01	0.35±0.08	0.07±0.17	−0.42±0.14	0.05±0.19
BW	0.49±0.02	0.22±0.02	0.15±0.02	0.28±0.06	0.24±0.15	0.09±0.17
HH	0.11±0.02	−0.01±0.02	−0.03±0.02	0.42±0.02	0.38±0.08	−0.17±0.21
SC	0.21±0.04	−0.02±0.07	0.02±0.07	0.39±0.04	0.20±0.05	0.40±0.10

LMA = longissimus muscle area (cm^2^); BF = backfat thickness (mm); RF = rump fat thickness (mm); SC = standardized scrotal circumference (mm) at 450 days; BW = body weight (kg); HH = hip height (cm).

**Table 3:** Estimates of heritability (diagonal), genetic (above diagonal) and residual (below diagonal) correlations, with standard errors, from model FA2G (model with two factors only for the matrix of additive genetic loading factors and in the residual matrix it was considered as in the standard multi-trait mixed models).

Traits	LMA	BF	RF	BW	HH	SC
LMA	0.25±0.05	0.10±0.12	0.07±0.10	0.03±0.08	−0.08±0.11	0.03±0.08
BF	0.14±0.02	0.31±0.08	0.31±0.13	−0.01±0.14	−0.33±0.12	0.08±0.15
RF	0.11±0.02	0.58±0.01	0.23±0.06	0.00±0.11	−0.25±0.12	0.05±0.12
BW	0.49±0.02	0.22±0.02	0.15±0.02	0.22±0.05	0.03±0.12	0.01±0.07
HH	0.11±0.02	−0.01±0.02	−0.03±0.02	0.42±0.02	0.30±0.08	−0.07±0.13
SC	0.21±0.04	−0.02±0.07	0.01±0.07	0.39±0.04	0.20±0.05	0.40±0.11

LMA = longissimus muscle area (cm^2^); BF = backfat thickness (mm); RF = rump fat thickness (mm); SC = standardized scrotal circumference (mm) at 450 days; BW = body weight (kg); HH = hip height (cm).

**Table 4:** Estimates of heritability (diagonal), genetic (above diagonal) and residual (below diagonal) correlations, with standard errors, from model REC1 (six recursive effects zeroed-out at residual covariance matrix).

Traits	LMA	BF	RF	BW	HH	SC
LMA	0.25±0.05	0.20±0.19	0.09±0.19	0.43±0.14	−0.16±0.17	0.25±0.20
BF	0.14±0.02	0.45±0.09	0.65±0.10	0.05±0.18	−0.49±0.13	0.05±0.23
RF	0.08±0.01	0.57±0.01	0.35±0.08	0.12±0.17	−0.40±0.15	0.04±0.19
BW	0.49±0.02	0.22±0.02	0.13±0.01	0.31±0.07	0.38±0.15	0.12±0.19
HH	0.11±0.02	0.00±0.02	−0.03±0.02	0.42±0.02	0.44±0.09	−0.19±0.23
SC	0.20±0.02	0.09±0.01	0.05±0.01	0.40±0.04	0.17±0.02	0.49±0.12

LMA = longissimus muscle area (cm^2^); BF = backfat thickness (mm); RF = rump fat thickness (mm); SC = standardized scrotal circumference (mm) at 450 days; BW = body weight (kg); HH = hip height (cm).

**Table 5:** Estimates of genetic (above the diagonal) and residual (below the diagonal) recursive effects, along with their standard errors, obtained from the FRM in Nellore cattle. Causal effects are specified from traits positioned to the left toward those on the right.

Traits	LMA	BF	RF	BW	HH	SC
LMA	—	0.40±0.40	−0.13±0.27	0.48±0.18	−0.54±0.27	0.23±0.48
BF	0.21±0.03	—	0.56±0.12	−0.08±0.12	−0.23±0.16	−0.09±0.32
RF	0.04±0.03	0.55±0.02	—	0.10±0.14	−0.24±0.19	−0.06±0.30
BW	0.45±0.02	0.09±0.02	0.01±0.02	—	0.83±0.25	0.34±0.49
HH	−0.14±0.03	−0.05±0.02	−0.04±0.02	0.60±0.03	—	−0.41±0.40
SC	0.03±0.06	−0.10±0.06	0.02±0.05	0.48±0.06	0.03±0.06	—

LMA = longissimus muscle area (cm^2^); BF = backfat thickness (mm); RF = rump fat thickness (mm); SC = standardized scrotal circumference (mm) at 450 days; BW = body weight (kg); HH = hip height (cm).

**Table 6:** Model comparison criteria to assess performance of different Bayesian models.

Models	DIC	$p_{D}$	Mean(L)
SMTM	37,874.7	456.5	**−18,709.2**
FA2F	38,036.8	455.2	−18,790.8
FA3F	38,915.2	564.1	−19,175.6
FA2G	37,898.3	431.2	−18,733.5
FA2R	38,043.5	499.7	−18,771.9
FRM	37,871.8	447.5	−18,712.1
REC1	37,868.6	443.6	−18,712.5
REC2	37,937.4	440.2	−18,748.6

SMTM = standard multi-trait mixed model; FA2F = model with two factors for additive genetic and residual loading matrices; FA3F = model with three factors for additive genetic and residual loading matrices; FA2G = model with two factors for additive genetic loading matrix only (residual as in SMTM); FA2R = model with two factors for residual loading matrix only (additive genetic as in SMTM); FRM = fully-recursive model; REC1 = model with six recursive effects zeroed-out at residual covariance matrix; REC2 = model with 10 recursive effects zeroed-out at residual covariance matrix and five at additive genetic covariance matrix. DIC = Deviance Information Criterion; $p_{D}$ = estimated number of effective parameters; Mean(L) = posterior mean of log-likelihood; The best value for each parameter (DIC; $p_{D}$; Mean(L)) is indicated in bold.

## Data Availability

The data used in this study can be made available upon request to the authors.

## Abbreviations

The following abbreviations are used in this manuscript:

BF	Backfat thickness
BLUP	Best Linear Unbiased Prediction
BOA	Bayesian Output Analysis
BW	Body weight
CGs	Contemporary groups
DIC	Deviance information criterion
EBV	Estimated breeding values
FA	Factor analytic model
FA3F	Model with three factors for genetic and residual matrices
FA2G	Model with two factors for the genetic matrix
FA2R	Model with two factors for residual matrix
FRM	Fully recursive model
HH	Hip height
LMA	Longissimus muscle area
MCMC	Markov Chain Monte Carlo
Mean(L)	Posterior mean of log-likelihood
MTM	Multivariate Gaussian Mixed Effects Model
$p_{D}$	Number of estimated parameters
REC	Recursive model
REC1	Model with six recursive effects zeroed out at the residual matrix
REC2	Model with 10 recursive effects zeroed-out at residual and five at genetic matrices
RF	Rump fat thickness
SC	Scrotal circumference
SEM	Structural equation model
SMTM	Standard multi-trait mixed model
